# Sleep and well-being before and after a shift schedule change in ICU nurses: an observational study using wearable sensors

**DOI:** 10.1093/joccuh/uiaf053

**Published:** 2025-09-19

**Authors:** Asami Ito-Masui, Ryota Sakamoto, Eiji Kawamoto, Eishi Motomura, Hisashi Tanii, Zachary D King, Kei Suzuki, Akane Sano, Motomu Shimaoka

**Affiliations:** Advanced Emergency and Critical Care Center, Mie University Hospital, 2-174 Edobashi, Tsu, Mie 514-8507, Japan; Department of Medical Informatics, Mie University Hospital, 2-174 Edobashi, Tsu, Mie 514-8507, Japan; Department of Molecular Pathobiology and Cell Adhesion Biology, Mie University Graduate School of Medicine, 2-174 Edobashi, Tsu, Mie 514-8507, Japan; Department of Neuropsychiatry, Mie University Graduate School of Medicine, 2-174 Edobashi, Tsu, Mie 514-8507, Japan; Center for Physical and Mental Health, Mie University, 1577 Kurimamachiya-cho, Tsu, Mie 514-8507, Japan; Department of Electrical and Computer Engineering, Rice University, 6100 Main Street, Houston, TX 77005-1827, USA; Advanced Emergency and Critical Care Center, Mie University Hospital, 2-174 Edobashi, Tsu, Mie 514-8507, Japan; Department of Electrical and Computer Engineering, Rice University, 6100 Main Street, Houston, TX 77005-1827, USA; Department of Molecular Pathobiology and Cell Adhesion Biology, Mie University Graduate School of Medicine, 2-174 Edobashi, Tsu, Mie 514-8507, Japan

**Keywords:** shift work sleep disorder, nurses, wearable devices, chronotype

## Abstract

**Objectives:** This study aimed to evaluate, using wearable sensors, the impact of transitioning from an 8-hour to a 12-hour shift schedule on sleep patterns and well-being in intensive care unit (ICU) nurses with pre-existing sleep disturbances. We also examined differences in outcome based on chronotype.

**Methods:** We conducted an observational study at a university hospital ICU between November 2020 and October 2023, before and after a hospital-wide shift schedule change. Nurses wore wearable sensors and completed daily surveys over 5 weeks under each shift system. Rotating-shift ICU nurses with a Pittsburgh Sleep Quality Index score >5 were eligible. Sleep metrics and subjective well-being were compared using linear mixed models, adjusting for age. Sleep episodes were categorized relative to shift timing, and chronotype-stratified subgroup analyses were performed.

**Results:** Eighty nurses completed the study (12-hour shift: 37; 8-hour shift: 43). The interval between shifts was greater for the 12-hour shift group (36.12 vs 26.78 hours). Total sleep duration did not significantly differ between groups (12-hour shift: 418.5 minutes; 8-hour shift: 398 minutes); however, the 12-hour shift group had less fragmented sleep, higher subjective well-being scores, and lower reported stress and fatigue. Evening chronotypes appeared to benefit more from 12-hour shifts, with longer sleep duration and higher well-being scores, though these differences were not statistically significant.

**Conclusions:** Transitioning to a 12-hour shift schedule was associated with reduced sleep fragmentation and improved well-being, particularly among evening chronotypes. These findings suggest that shift schedule structure and individual chronotype may influence adaptation to shift work in ICU settings.

## Teaser

**Key points**
 

- This is one of the first studies to combine wearable sensor data with daily self-reported well-being assessments over 35 days in a real-world ICU setting, evaluating the impact of transitioning from an 8-hour to a 12-hour shift schedule. Unlike most prior studies relying on surveys or short-term monitoring, our approach captured daily fluctuations in sleep and well-being, allowing for more stable and accurate evaluation of shift-related effects.
- Although 12-hour shifts did not increase total sleep duration, they were associated with reduced sleep fragmentation and lower social jet lag. These findings suggest that the distribution and regularity of sleep, rather than its total quantity, may be more linked to improved recovery, circadian alignment, and subjective well-being.
- These findings suggest that 12-hour shifts may improve sleep patterns and well-being in ICU nurses, particularly those with evening chronotypes, and support the consideration of longer shifts provided that sufficient inter-shift intervals are ensured.

## 1. Introduction

Shift workers play a vital role in health care settings where continuous 24-hour coverage is required. Globally, approximately 20% of the workforce engages in shift work. However, shift work leads to circadian misalignment, causing sleep disturbances and increased risk of mental health issues and burnout.^1^^,^^2^ Over time, this misalignment can lead to shift work sleep disorder, characterized by insomnia, excessive sleepiness, and cognitive impairment. Overall prevalence of shift work sleep disorder is reported to be 26.5%,^3^ with prevalence rates of insomnia ranging from 12% to over 70%, depending on the population.^4^ Insufficient sleep related to night shifts impairs cognitive performance in intensive care unit (ICU) nurses,^5^ and is also considered to contribute to medication errors.^6^ In addition, multiple studies confirm that sleep problems, such as insomnia, significantly increase the risk and severity of burnout syndrome in nurses.^7^^,^^8^

The 12-hour shift model is increasingly adopted in hospitals worldwide. A 2014 national survey in the United States reported that 65% of registered nurses worked 12 to 13 hours, and 80% of ICU nurses worked 12 to 13 hours on their last shift.^9^ Debate continues over the optimal shift schedule, yet longer shifts have become more prevalent over the past decades. Direct evidence on how this transition affects objective sleep patterns, particularly in high-intensity environments like the ICU, remains limited. In questionnaire-based studies, nurses often report preferring 12-hour shifts due to fewer handovers and more days off.^10^^,^^11^ Although some studies have reported longer sleep duration for 12-hour shifts compared with 8-hour shifts,^12^ systematic reviews suggest that 12-hour shifts do not consistently improve sleep outcomes across populations.^13^ Evaluating this change in shift patterns using wearable sensor data provides a rare opportunity to capture its real-world impact with high ecological validity. Wearable devices are increasingly used to assess sleep and circadian disruption in real-world shift work settings, offering a practical alternative to laboratory-based methods.^14^

Moreover, few studies have assessed how chronotypes, increasingly recognized as a key moderator of shift tolerance, interact with different shift lengths in real clinical settings. Evening chronotypes may adapt better and prefer night shifts,^15^ compared with morning types. Understanding this relationship is essential for developing a personalized shift schedule that minimizes sleep disruption and promotes staff well-being.

This study aimed to assess the impact of transitioning from an 8-hour to a 12-hour shift schedule on sleep outcomes using wearable sensors among ICU nurses with pre-existing sleep disturbances, as well as subgroup analyses by chronotype, to assess how shift structure and individual circadian preferences interact to shape health outcomes in ICU nurses.

## 2. Methods

### 2.1. Study design

This was a pre–post observational study using data from a prior intervention study. In December 2021, the hospital transitioned its shift schedule for ICU nurses from 8-hour to 12-hour shifts. Nurses working under the original 8-hour schedule and those under the new 12-hour schedule comprised the 2 cohorts. Both groups participated in the same sleep improvement intervention, which began after a 1-week baseline. We compared the sleep and well-being of the 2 groups using wearable sensors and daily surveys over 5 weeks.

### 2.2. Participants and setting

This study was conducted in a 26-bed ICU at a university hospital. Participants were eligible if they were nurses on a rotating shift schedule with at least 1 night shift per week and had a Pittsburgh Sleep Quality Index (PSQI) score greater than 5.^16^ This criterion targeted individuals with mild or greater sleep disturbances, as they represent a population that requires support. Participants who were pregnant or receiving treatment for psychiatric disorders were excluded, as these conditions could independently affect sleep patterns. All participants were required to participate in a sleep improvement intervention delivered via a mobile application. Following a 1 week baseline data collection, the participants received 4 weeks of the program, which included professionally developed sleep advice.^17^ Data sampling for the 12-hour shift group started 1 year after the implementation of the new shift schedule to allow time for adjustment. Both shift schedules employed rapid rotation, with nurses switching between different shift types within a few days. The 12-hour shift schedule consisted of a day shift (8:00 am to 8:00 pm) and a night shift (8:00 pm to 8:00 am), whereas the 8-hour shift schedule included a day shift (8:00 am to 4:00 pm), evening shift (4:00 pm to midnight), and night shift (midnight to 8:00 am). Quick returns were common under the 8-hour schedule, with examples including a night shift following a day shift with an 8-hour gap. The 12-hour schedule did not allow consecutive night shifts on the same day; nurses had a minimum of 12 hours between shifts, and at least 36 hours between 2 night shifts. Off-days were scheduled monthly with their timing varying based on staffing needs and individual requests. On average, nurses had 9.3 off-days per month in the 12-hour shift group and 6.5 in the 8-hour shift group. Data for the 8-hour shift were collected from November 2020 to October 2021, and for the 12-hour shift from November 2022 to October 2023. Participants were recruited via e-mail and flyers using convenience sampling, then screened for eligibility. Of the 86 screened, 82 enrolled, and 2 dropped out due to low participation and nonadherence to the protocol. A total of 80 participants completed the study. They received monetary rewards after the study period.

### 2.3. Outcome and measurements

#### 2.3.1. Outcomes

The primary outcome of this study was the comparison of daily sleep variables between 8-hour and 12-hour shift schedules. These variables included total time in bed, number of sleep episodes per day, sleep efficiency, and the Sleep Regularity Index (SRI). Secondary outcomes included: (1) daily subjective well-being scores and related measures (eg, perceived stress and fatigue); (2) sleep episode–level comparison, grouped by sleep timing categories (eg, before/after night shift), of variables including total time in bed, mid-sleep timing, and social jet-lag; (3) subgroup analyses based on chronotype; and (4) the association between sleep parameters and well-being scores.

#### 2.3.2. Wearable sensor data and sleep parameters

Fitbit Charge 3 (Fitbit; Google, Menlo Park, CA, USA) was used to measure sleep-related variables, steps, and mean heart rate. Participants were instructed to wear the device 24 hours a day, except when charging, bathing, or for work-related reasons. Direct sleep measurements obtained from the Fitbit export included the total time in bed, total minutes asleep, total minutes awake, number of sleep episodes per day, sleep onset and offset times, and classification of each episode as main or short sleep. Main sleep was defined as the longest sleep period of the day, whereas short sleep (naps or secondary sleep) referred to other sleep episodes during the same day. The Fitbit assigned sleep to the date on which it ended.

Based on these exported data, we calculated additional sleep metrics: sleep efficiency (total minutes asleep divided by total time in bed), mid-sleep time (midpoint between sleep onset and offset), social jet lag (difference in mid-sleep time between free days and workdays),^18^ and the SRI, calculated by summing the number of matching 30-second sleep–wake epochs across consecutive days over the 35-day study period.^19^

#### 2.3.3. Questionnaires and daily surveys

The assessments used in this study were divided into pre-study questionnaires and daily surveys. The pre-study questionnaires included the PSQI; 12-item General Health Questionnaire^20^; Japan Burnout Scale, based on the Maslach Burnout Inventory^21^; State–Trait Anxiety Inventory (STAI)^22^; and Morningness-Eveningness Questionnaire (MEQ).^23^ The MEQ was used to categorize participants based on chronotype. We categorized the participants as morning type [combining “definitely morning type (86-70)” and “moderately morning type (69-59)”], evening type [combining “definitely evening type (30-16)” and “moderately evening type (41-31)”], and neither type (58-42). Permission to use the STAI was obtained from the copyright holder at Sankyobo (Kyoto, Japan).

Daily surveys were administered twice at 6:00 am (morning survey) and 6:00 pm (evening survey). Morning surveys included morning well-being scores, whereas evening surveys included evening well-being scores, stress and fatigue levels, and daily activities and habits (eg, alcohol and caffeine intake). Well-being was measured as the sum of 5 components—happiness, alertness, health, energy, and relaxation—each rated on a 0-100 score. This composite score was originally developed by our study group and used in previous work to assess and predict well-being.^24^ Participants provided well-being scores using a digital visual analog scale. Stress and fatigue levels were rated from 1 to 5, with lower scores indicating higher levels of stress or fatigue. These repeated daily measures, along with wearable sensor data, were later analyzed using mixed-effects models. Participants also recorded their work status in 30-minute increments, including additional hours worked beyond their scheduled shifts. The recorded work hours were used to calculate average working hours and intervals between shifts, and to distinguish shift timing (day shift, night shift, off days).

### 2.4. Statistical analyses

This study represents a secondary analysis of data originally collected for a sleep intervention study. Power calculations for the primary study, based on pilot data, estimated that 66 participants would be required to detect a 30-minute difference in sleep duration, assuming an SD of 135.1 min, 80% power, and a 5% significance level (2-sided) using a paired *t* test. However, the present analysis compared 2 independent shift groups, and the original power analysis did not directly apply. Given institutional constraints, the maximum number of eligible participants available was recruited.

We conducted 2 levels of analysis to compare sleep and well-being between 8-hour and 12-hour shift schedules: (1) Daily-level analysis: Sleep variables included total time in bed, sleep efficiency, and the SRI. The total time in bed was the sum of all sleep episodes when there were multiple sleep episodes in 1 day. We compared the total time in bed and other wearable metrics, along with daily well-being scores, daily fatigue scores, and other daily repeated measurements between 12-hour and 8-hour shifts. (2) Episode-level analysis: Each sleep episode was treated as an independent observation. Sleep episodes were categorized into 5 groups relative to shift timing: sleep after a night shift, sleep before a night shift, sleep before a day shift, sleep on free days, and all other sleep types. We then compared the total sleep duration (including and excluding short sleep episodes), the number of short sleep episodes, and mid-sleep time across these categories.

For descriptive statistics, continuous variables are expressed as medians and interquartile ranges, and categorical variables as counts and percentages. For inferential analysis, linear mixed models (LMMs) were used for repeated continuous outcomes, with shift type as a fixed effect and participant ID as a random intercept to account for within-subject variability. For categorized variables, a generalized linear mixed model with logit link function was used, with estimates and 95% CIs reported on the log-odds scale. Raw *P* values were calculated using the Satterthwaite approximation, and statistical significance was defined as 2-sided *P* < .05. Bonferroni-corrected *P* values were reported to account for multiple comparisons. For chronotype subgroup analyses, an LMM was used with shift group, chronotype, and age as fixed effects and participant ID as a random effect, with the 8-hour shift and morning type serving as the reference group.

Participants were included in the analysis if they had at least 4 valid days (>60% of daily wear time^25^) out of 7. This study was a secondary analysis of data from a prior study in which both shift groups underwent the same intervention, following a 1-week baseline. As a sensitivity analysis, we repeated the primary comparisons using only baseline data. The results were consistent with those from the full 5-week dataset. Missing data were handled by excluding incomplete cases. All analyses were conducted using R version 4.4.1(R Foundation for Statistical Computing, Vienna, Austria).

### 2.5. Ethical considerations

This study and the original intervention study were approved by the Ethics Committee of Mie University (approval numbers: H2022-134, H2020-083) and conducted in accordance with the ethical guidelines for human research. All participants provided written informed consent. This study was registered with the Japan Registry of Clinical Trials (trial number: CRB4180006).

## 3. Results

### 3.1. Participant characteristics

Thirty-seven and 43 participants were included in the 12-hour and 8-hour shift groups, respectively (Table 1). Fifteen participants were included in both shift groups. A total of 2798 days were measured, 1296 days for the 12-hour shift, and 1502 days for the 8-hour shift. There were fewer night shifts (552 days vs 816 days) and more off-days (427 days vs 356 days) under the 12-hour shift system. The 12-hour shift had a higher proportion of moderately evening chronotypes. During the study period, patient numbers were higher in the 12-hour shift, whereas ICU bed occupancy was higher in the 8-hour shift. Baseline demographics and questionnaire scores for psychological and sleep evaluation did not differ between the groups. Whereas the average work hours per day were similar between the 2 shifts, the interval between shifts was greater for the 12-hour shift (36.12 vs 26.78 hours). The proportion of participants providing >30 days of wearable sensor data was 86.3%.

**Table 1:** Participant characteristics of 12-hour and 8-hour shift groups.

	**12-hour shift (*n* = 37)**	**8-hour shift (*n* = 43)**	**SMD**
***Demographics of participants and work***			
**Age, mean [SD], y**	31 [27-37]	29 [26-37]	0.119
**Sex, female/male, *n* (%)**	32/5 (86.5/13.5)	37/6 (86.0/14.0)	0.013
**MEQ, *n* (%)**			
**Definitely morning type**	0 (0)	0 (0)	
**Moderately morning type**	3 (8.2)	6 (13.9)	−0.185
**Neither type**	27 (72.9)	34 (79.1)	−0.143
**Moderately evening type**	7 (18.9)	1 (2.3)	0.553
**Definitely evening type**	0 (0)	2 (4.7)	−0.298
**ICU patients per day, median [IQR]**	16 [14–17]	14 [12–16]	0.742
**ICU bed occupancy rate, median [IQR], %**	65.7 [58.3–70.8]	70.0 [60.0–80.0]	0.359
**ER patients per day, median [IQR]**	4 [3–6]	3 [2–5]	0.447
**Number of shifts, *n* (%), d**			
**Total days**	1296	1502	
**Day shift**	317 (24.4)	330 (22.0)	0.059
**Night shift**	552 (42.6)	816 (54.3)	0.236
**Off**	427 (33.0)	356 (23.7)	0.441
**Work hours, mean [SD], h**	5.95 [4.95]	5.73 [3.91]	0.049
**Intervals between shifts, median [IQR],h**	36.1 [34.6–40.4]	26.8 [24.7–28.2]	2.588
**Alcohol consumption, median [IQR], cups**	0 [0–0]	0 [0–0]	0.020
**Caffeine consumption, median [IQR], cups**	0 [0–1]	0 [0–1]	0.021
***Pre-study questionnaire***			
**PSQI, median [IQR]**	8 [6–9]	8 [7–11]	−0.477
**STAI-S, median [IQR]**	44.0 [39.0–47.0]	44 [37.5–52.0]	−0.138
**STAI-T, median [IQR]**	53 [48–57]	51 [42–55]	0.385
**GHQ, median [IQR]**	9 [7–10]	7 [5–10]	0.293
**JBS-e, median [IQR]**	3.6 [3.0–4.0]	3.8 [3.2–4.2]	−0.289
**JBS-d, median [IQR]**	2.2 [1.5–2.5]	2.0 [1.7–2.7]	−0.200
**JBS-pa, median [IQR]**	3.8 [3.3–4.0]	3.8 [3.3–4.3]	−0.158

Abbreviations: ER, emergency room; GHQ, 12-item General Health Questionnaire; ICU, intensive care unit; IQR, interquartile range; JBS-e/d/pa, Japan Burnout Scale—e/d/pa, emotional exhaustion, depersonalization, personal achievement; MEQ, Morningness–Eveningness Questionnaire; PSQI, Pittsburgh Sleep Quality Index; SMD, standardized mean difference; STAI-S/T, State Trait Anxiety Inventory—State/Trait.

### 3.2. Daily sleep and well-being outcome comparison between shift schedules

Wearable data showed no significant differences in total time in bed between the 12-hour and 8-hour shift groups. The median values were 418.5 minutes and 398.0 minutes, respectively (Table 2). The LMM confirmed that shift type was not significantly associated with total time in bed (estimate: −10.35 minutes; 95% CI, −32.60 to 11.91; adjusted *P* value: 1.000). However, the 8-hour shift had more sleep episodes per day than the 12-hour shift (estimate: 0.11; 95% CI, 0.03 to 0.19; adjusted *P* value: .083). Other sleep-related variables, including sleep efficiency and SRI, showed no significant differences between the groups. Both morning and evening well-being scores were higher in the 12-hour shift group than in the 8-hour shift group. Additionally, participants in the former group reported lower levels of stress and fatigue than those in the 8-hour shift group.

**Table 2:** Comparison of daily measurements between 12-hour and 8-hour shifts.

	**12-hour shift (*n* = 37)**	**8-hour shift (*n* = 43)**	**Estimate**	**95% CI**	**Raw *P* value**	**Adjusted *P* value**
***Sleep and other biological parameters***						
**Total time in bed, median [IQR], min**	418.5 [310.0 to 501.3]	398.0 [281.0 to 498.0]	−10.35	[−32.60 to 11.91]	.365	1.000
**Sleep episodes per day, median [IQR]**	1 [1 to 2]	1 [1 to 2]	0.11	[0.03 to 0.19]	.006	.083
**Sleep efficiency, median [IQR]**	95 [92 to 97]	95 [92 to 97]	−0.13	[−1.04 to 0.77]	.777	1.000
**Steps, median [IQR]**	7654.0 [3991.0 to 11791.0]	7175.0 [3933.3 to 10289.5]	−351.55	[−141.89 to 0.85]	.590	1.000
**Mean heartrate, median [IQR], bpm**	74.6 [68.0 to 80.3]	74.1 [67.9 to 80.6]	2.658	[−1.63 to 6.94]	.228	1.000
***Well-being and stress parameters***						
**Morning well-being score, median [IQR], 0-500**	250.0 [205.0 to 319.0]	222.5 [156.0 to 273.3]	−37.38	[−62.94 to −11.80]	.005	.069
**Evening well-being score, median [IQR], 0-500**	250.0 [204.0 to 312.3]	226.0 [161.3 to 280.0]	−31.9	[−56.29 to −7.50]	.012	.156
**Daytime sleepiness, median [IQR], 0-10**	6 [4 to 7]	6 [3 to 7]	−0.10	[−0.34 to 0.12]	.363	1.000
**Feeling of stress, median [IQR], 0-5**	3 [2 to 4]	3 [2 to 4]	−0.33	[−0.61 to −0.06]	.016	.208
**Feeling of fatigue, median [IQR], 0-5**	2 [2 to 3]	2 [1 to 3]	−0.26	[−0.49 to −0.03]	.024	.312
**Subjective sleep quality, median [IQR], 0-25**	16 [12 to 19]	15 [11 to 18]	−1.13	[−2.40 to 0.13]	.084	1.000
**PVT morning, median [IQR], s**	338.0 [267.5 to 393.5]	332.0 [0.0 to 386.0]	−23.23	[−76.58 to 30.13]	.396	1.000
**PVT evening, median [IQR], s**	339.0 [280.0 to 400.0]	330.0 [0.0 to 388.0]	−70.52	[−141.89 to 0.85]	.056	.728

Abbreviations: IQR, interquartile range; PVT, psychomotor vigilance test.

### 3.3. Sleep episode–level comparison of sleep variables between 12- and 8-hour shift schedules

Our analysis revealed that the 8-hour shift group had significantly more short sleep episodes after and before night shifts than the 12-hour shift group (Table 3). The total time in bed after a night shift was statistically significantly longer in the 8-hour shift group than in the 12-hour shift group (adjusted *P* value: .001). Before a night shift, the total time spent in bed was longer in the 8-hour shift group when short sleep episodes were excluded, but shorter when they were included. Mid-sleep timing before and after night shifts was more variable in the 8-hour shift group than in the 12-hour shift group, indicating greater inconsistency in sleep patterns. Social jet lag, defined as the absolute difference in mid-sleep timing between workdays (day shift) and free days, was greater in the 8-hour shift group (4 hours 8 minutes) compared with the 12-hour shift group (3 hours 33 minutes), suggesting greater circadian misalignment.

**Table 3:** Sleep episode characteristics by shift-related timing in 12-hour vs 8-hour shifts.

	**12-hour shift**	**8-hour shift**	**Estimate**	**95% CI**	**Raw *P* value**	**Adjusted *P* value**
**Number of sleep records**						
**Total episodes**	1495	1885				
**After night shift**	205	730				
**Before night shift**	441	413				
**Before day shift**	311	273				
**Free day**	487	459				
**Other**	51	10				
**Number of short sleeps, *n* (%)**						
**After night shift**	22 (10.7)	189 (25.9)	1.08	[0.57 to 1.58]	<.001	<.001
**Before night shift**	180 (40.8)	219 (53.0)	0.49	[0.22 to 0.76]	<.001	.001
**Before day shift**	2 (0.6)	9 (3.3)	1.47	[−1.44 to 4.39]	.322	1.000
**Free day**	107 (22.0)	125 (27.2)	0.28	[−0.14 to 0.70]	.191	.764
**Sleep duration, min (including short sleeps)**						
**After night shift, mean (SD)**	213.9 (109.5)	268.8 (154.7)	61	[30.38 to 91.61]	<.001	.001
**Before night shift, mean (SD)**	367.7 (204.2)	333.2 (212.9)	−37.13	[−72.28 to −1.98]	.042	.336
**Before day shift, mean (SD)**	392.3 (91.0)	383.5 (106.7)	−12.18	[−40.74 to 16.37]	.406	1.000
**Free day, mean (SD)**	387.9 (181.1)	372.0 (189.2)	−5.66	[−41.67 to 30.34]	.758	1.000
**Sleep duration, min (main sleep only)**						
**After night shift, mean (SD)**	225.2 (108.0)	318.9 (146.6)	96.38	[66.30 to 126.44]	<.001	<.001
**Before night shift, mean (SD)**	499.9 (155.7)	510.7 (177.0)	9.03	[−36.22 to 54.29]	.697	1.000
**Before day shift, mean (SD)**	394.1 (88.8)	391.7 (98.4)	−6.74	[−34.12 to 20.64]	.631	1.000
**Free day, mean (SD)**	459.9 (132.1)	458.6 (140.6)	2.45	[−26.81 to 31.72]	.87	1.000
**Mid sleep, h:min:s**						
**After night shift, mean (SD)**	14:54:34 (02:41:49)	11:27:54 (05:31:50)	−03:28:15	[02:30:25 to 04:26:04]	<.001	<.001
**Before night shift, mean (SD)**	09:30:53 (05:24:14)	12:35:17 (07:50:43)	03:11:19	[−05:45:00 to −03:52:20]	<.001	<.001
**Before day shift, mean (SD)**	03:17:20 (01:28:12)	03:32:56 (02:35:18)	00:16:07	[−01:18:04 to 00:09:41]	.225	1.000
**Free day, mean (SD)**	06:50:38 (04:58:36)	07:40:07 (06:04:47)	00:43:20	[−02:19:33 to 00:13:46]	.141	1.000

### 3.4. Subgroup analysis of sleep and well-being by chronotypes

Evening-type participants of the 12-hour shift schedule spent the longest time in bed (431.31 minutes), whereas morning-type participants on the same schedule spent the shortest (376.49 minutes). Regarding well-being scores, morning-type nurses in the 12-hour shift group had the highest scores, whereas evening-type nurses in the 8-hour shift group had the lowest scores (Table S1). Although the neither-type and the evening-type groups spent more time in bed than the 8-hour shift morning-type group (the reference group), LMM results did not show significant differences. Similarly, the 12-hour shift and evening-chronotype combinations were associated with higher well-being scores compared with the reference group, although this was not statistically significant (Table S2).

### 3.5. Association between well-being and total sleep time

**Figure 1:**
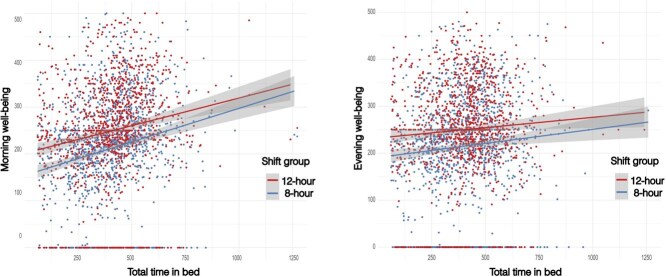
Correlation between total time in bed and well-being. The plot shows the correlation between the total time spent in bed and morning well-being (left) and evening well-being (right) of the two shift schedules (12-hour and 8-hour ). The morning well-being and total time in bed showed a weak correlation for both 12- and 8-hour shifts, with Pearson correlation coefficients of 0.171 and 0.203, respectively. Almost no correlation was observed for evening well-being, with Pearson correlation coefficients of 0.067 and 0.0842, respectively.

As shown in Figure 1, the total time in bed showed a weak positive correlation with morning well-being in both 12-hour and 8-hour shift groups (Pearson correlation coefficients 0.17 and 0.20, respectively). LMM confirmed that this association was statistically significant for both the 12-hour shift group (estimate = 0.12; SE = 0.018; adjusted *P* < .01) and the 8-hour shift group (estimate = 0.12; SE = 0.017; adjusted *P* < .01). No linear correlation was observed for evening well-being and total time in bed (Pearson correlation coefficients 0.067 and 0.084, respectively). However, the LMM for evening well-being showed that the association was significant in the 12-hour shift group (estimate = 0.037; SE = 0.017; adjusted *P* = .136) but not significant in the 8-hour shift group (estimate = 0.026; SE = 0.017; adjusted *P* = .520). No significant associations were observed between well-being and other sleep variables. To further examine differences between day and night shifts, we evaluated the associations between total time in bed and well-being, stratified by shift type (day shift and night shift). Similarly, the results showed a weak positive correlation between total sleep time and morning well-being, whereas no clear correlation was observed with evening well-being (Figure S1), for both day and night shifts.

## 4. Discussion

This study is among the first to use both wearable sensor data and daily self-reported well-being assessments in a real-world clinical setting to evaluate the impact of transitioning from an 8-hour to a 12-hour shift schedule in ICU nurses with pre-existing sleep disturbances. Unlike most previous studies that relied solely on questionnaires or short observation windows, we assessed 30 days of continuous data per participant and accounted for chronotype differences—factors often neglected in previous shift work research.

A key novel finding is that total sleep duration did not differ significantly between the 2 shift schedules, yet sleep fragmentation and circadian disruption were lower in the 12-hour group. Although not statistically significant, the 12-hour shift group showed slightly longer total time in bed, which may reflect a greater need for recovery due to longer work hours. Paradoxically, when analyzing each sleep episode, we found that sleep following night shift was significantly shorter in the 12-hour shift group. Although speculative, one possible explanation is that preserved circadian alignment in this group made daytime sleep more difficult following night shifts. In contrast, they may have had more opportunities to sleep during their biological night in the other sleep categories—such as sleep before night shifts, before day shifts, and on off-days. Notably, nurses on 8-hour shifts exhibited more frequent short sleep episodes and greater variability in sleep timing, particularly around night shifts. This aligns with existing evidence that evening shifts followed by day shifts can lead to restricted and less restorative sleep.^26^ Although short naps can be restorative under specific conditions,^27^ chronic sleep fragmentation can impair alertness and exacerbate sleep inertia.^28^ These findings highlight that not only the amount, but also the distribution and quality of sleep, are critical for recovery and performance.

Although nurses on the 12-hour shift had lower sleep duration after night shifts, their subjective well-being scores were higher, with lower reported stress and fatigue. Similar reports have shown improvements in burnout levels^29^^,^^30^ and fatigue^30^ related to 12-hour shifts. Our results also indicated a reduced circadian misalignment in the 12-hour shift group, which may partly explain improvements in both subjective sleep quality and well-being. Although some studies suggest that rotational 12-hour shifts may increase social jet lag,^31^ the present study found that the 12-hour schedule was associated with less social jet lag and more regular sleep timing. As described earlier, the 8-hour shift accommodated quick returns, whereas the 12-hour shift group had larger intervals between shifts. Additionally, the 12-hour shift group had more off-days, reflecting the concentrated work schedule. These results suggest that sufficient recovery time between shifts may counterbalance the demands of longer work hours, leading to improved fatigue and well-being. These differences in the 12-hour shift structure may explain the discrepancies with past studies that report increased fatigue and poor sleep in 12-hour shifts.^32^^,^^33^

In the present study, we found a weak correlation between the total sleep duration and morning well-being. Although our study did not directly assess whether the change in sleep patterns affected the well-being of shift workers, randomized controlled studies have shown that improving insomnia in shift workers’ sleep disorders also improves their anxiety and depression levels.^34^^,^^35^ Taken together, we can suspect that improvement in perceived sleep satisfaction and less sleep disruption may have played a part in the improvement of subjective daily well-being. These findings indicate that the structure and spacing of shifts may mitigate these negative effects of longer shift durations.

In our comparison of chronotypes, participants in the 12-hour shift schedule showed an increase in sleep hours and well-being scores among those with an evening chronotype. In contrast, total sleep duration decreased in morning-chronotype participants. Because evening-chronotype individuals naturally prefer staying awake and waking later, the extended rest periods in a 12-hour shift may better align with their biological rhythms, allowing for longer and more consolidated sleep. Additionally, the longer duration between shifts may help them extend their sleep into the late morning or afternoon, when they naturally sleep longer. A previous study found that increased eveningness was associated with longer main sleep episodes during consecutive night shifts.^36^ Evening chronotypes also achieve a higher percentage of sleep requirements before starting a night shift than morning chronotypes.^37^ Conversely, morning chronotypes may struggle to maintain early bedtime habits when working long shifts, leading to reduced sleep. Since early chronotypes tend to fall asleep approximately an hour earlier than late chronotypes on morning shifts, late work-end times can make it particularly difficult for them to maintain their sleep schedules.^38^ Studies have led to the recognition that accounting for individual chronotypes is important when designing shift schedules. However, the present findings should be interpreted with caution, as we did not directly compare evening types across shift schedules, and the small sample size and uneven chronotype distribution may have influenced the results.

Although this study provides valuable insights, there are several limitations that must be considered. First, as an observational study based on institutional implementation, the systematic differences between groups cannot be fully controlled. For example, the 12-hour shift group had longer inter-shift intervals and more days off, with the 8-hour shift group having more quick returns, which may have independently contributed to improved sleep and well-being. As discussed above, these structural differences may help explain the observed outcomes, making it difficult to isolate the effect of shift length alone. Additionally, there were more evening chronotypes in the 12-hour group, who may have naturally tolerated night shifts better. The timing of data collection also differed, with the 8-hour group’s data partially overlapping with the COVID-19 pandemic, potentially impacting stress levels; however, we attempted to minimize these effects by temporarily pausing data collection during the COVID-19 pandemic, during which the Japanese government issued a state of emergency. In addition, we used statistical adjustments and sensitivity analyses to mitigate some of these effects. Nonetheless, the findings should be interpreted with caution.

Another limitation is the use of consumer wearables for data collection. Although they are widely used in real-world sleep research, Fitbit may fail to detect short sleep episodes, particularly those under 1 hour,^39^ and show larger discrepancies from polysomnography with shorter sleep duration.^40^ Next, since we used convenience sampling in a single hospital, the results may not be generalizable to other professions or health care settings. Finally, the data involved a sleep intervention study, which may alter sleep conditions. However, since all participants uniformly received the same sleep intervention during the study period, this is unlikely to have confounded the comparison between shift types. Furthermore, sensitivity analyses using only baseline (pre-intervention) data produced similar results, supporting the robustness of our findings. Future studies should investigate not only the length of shifts but also the spacing between them, as both factors likely interact to influence sleep and overall well-being. In particular, studies that incorporate objective sleep data, real-world scheduling variability, and individual chronotype differences will be essential for designing shift schedules that support both clinical performance and worker resilience within health care.

## 5. Conclusion

In conclusion, transitioning from an 8-hour to a 12-hour shift does not significantly affect total sleep time but is associated with reduced sleep fragmentation and improved well-being. Although this study suggests that a 12-hour shift schedule may be more beneficial in terms of sleep and well-being, previous studies have reported different findings under varying shift structures. The influence of shift duration should be interpreted in the context of broader scheduling structures.

## Supplementary Material

Web_Material_uiaf053

## Data Availability

Data are available upon reasonable request.
