# Supplementary figures and images for: Sleep and well-being before and after a shift schedule change in ICU nurses: an observational study using wearable sensors
Source: J Occup Health. 2025 Sep 19;67(1):uiaf053. doi: 10.1093/joccuh/uiaf053 (PMC12481240; doi:10.1093/joccuh/uiaf053)

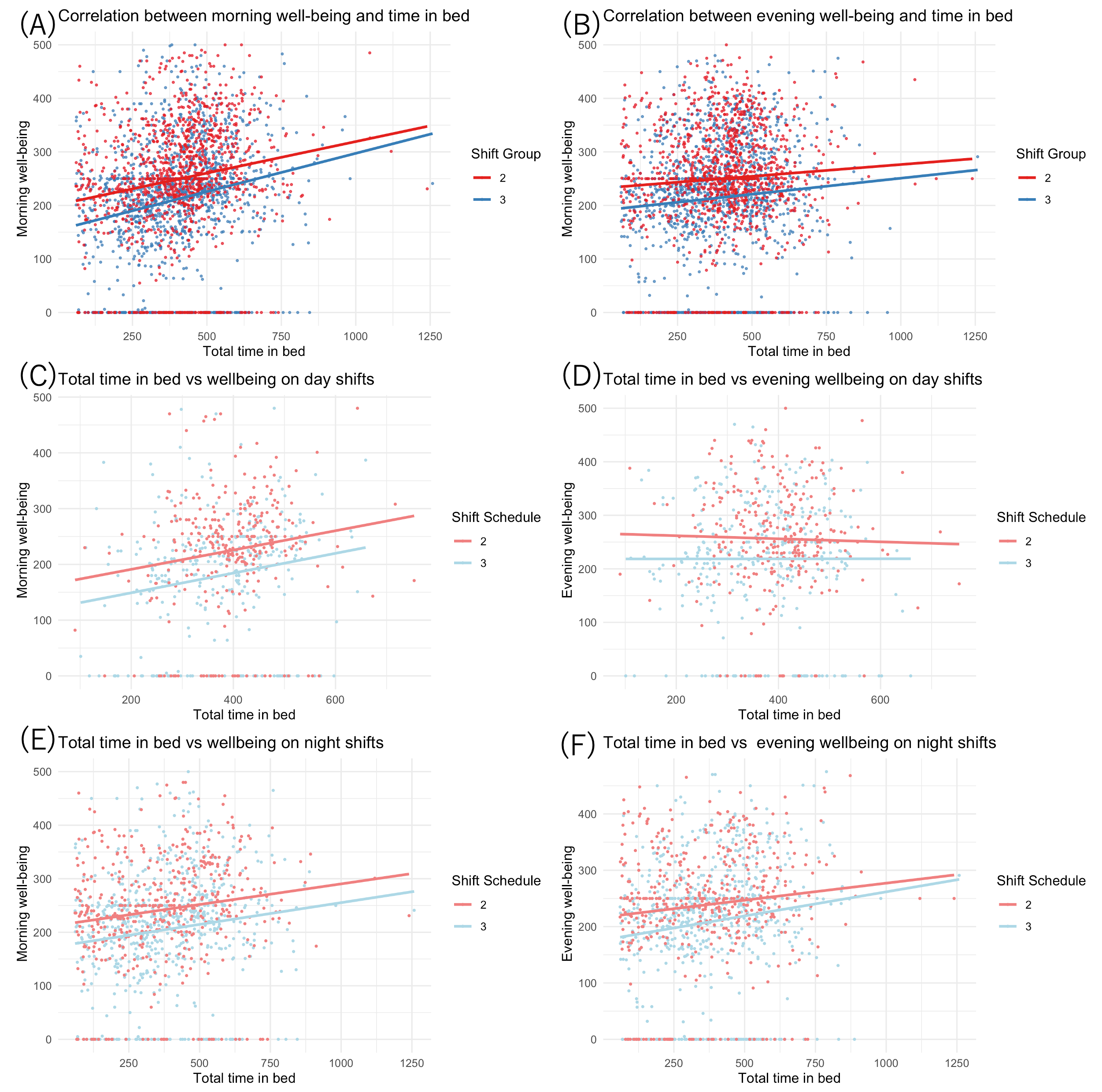

Supplement: Web_Material_uiaf053 [file web_material_uiaf053.zip › supplementary figure 1.png]
